# Supplementary material for: Coherent transport through a Majorana island in an Aharonov–Bohm interferometer
Source: Nat Commun. 2020 Jun 25;11:3212. doi: 10.1038/s41467-020-16988-x (PMC7316771; doi:10.1038/s41467-020-16988-x)
Supplement: Supplementary file 1 — Supplementary Information [file 41467_2020_16988_MOESM1_ESM.pdf]

Supplementary Information

Coherent transport through a Majorana island in an Aharonov-Bohm interferometer

A. M. Whiticar,[1] A. Fornieri,[1] E. C. T. O'Farrell,[1] A. C. C. Drachmann,[1] T. Wang,[2,3] C. Thomas,[2,3] S. Gronin,[2,3] R. Kallaher,[2,3] G. C. Gardner,[2,3] M. J. Manfra,[2,3,4,5] C. M. Marcus,[1] and F. Nichele[1]

[1] *Center for Quantum Devices, Niels Bohr Institute, University of Copenhagen and Microsoft Quantum Lab Copenhagen, Universitetsparken 5, 2100 Copenhagen, Denmark*

[2] *Department of Physics and Astronomy and Microsoft Quantum Lab Purdue, Purdue University, West Lafayette, Indiana 47907 USA*

[3] *Birck Nanotechnology Center, Purdue University, West Lafayette, Indiana 47907 USA*

[4] *School of Materials Engineering, Purdue University, West Lafayette, Indiana 47907 USA*

[5] *School of Electrical and Computer Engineering, Purdue University, West Lafayette, Indiana 47907 USA*

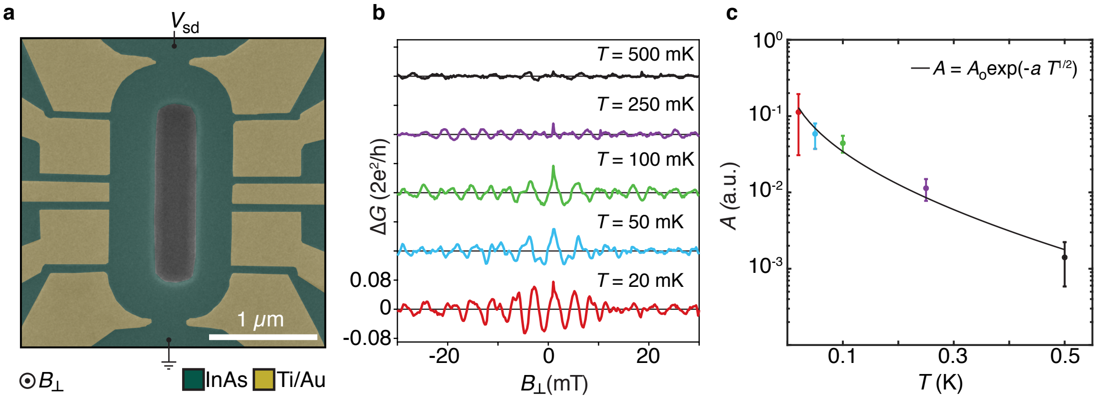

Supplementary Figure 1. **Temperature dependence of Aharonov-Bohm oscillations in a normal 2DEG.** **a**, False-colour electron micrograph of a normal conducting AB interferometer defined in an InAs 2DEG (green) by gates (Ti/Au). The hole forming the interferometer center is created by a wet etch. **b**, Magnetoconductance ΔG as a function of perpendicular magnetic field $B_\perp$ controlling the flux in the interferometer for several temperatures. Periodic oscillations are observed with a frequency of $f \sim 0.26\ \mathrm{mT}^{-1}$, which agrees with a single magnetic flux quantum h/e piercing the interferometer loop. **c**, Temperature dependence of the AB oscillations amplitude A measured from the power spectrum of the curves in **b**. For a diffusive interferometer, the amplitude $A = A_0 \exp(-L/l_\phi(T))$ where $l_\phi(T) \propto T^{-1/2}$ is the phase coherence length and $L = 4.5\ \mu$m is the circumference of the interferometer [1]. The exponential fit $A = A_0 \exp(-aT^{1/2})$ gives a base temperature coherence length of $l_\phi(20\ \mathrm{mK}) = 4\ \mu\mathrm{m} \pm 1\ \mu\mathrm{m}$. Error bars show the standard deviation between 4 data sets at each temperature.

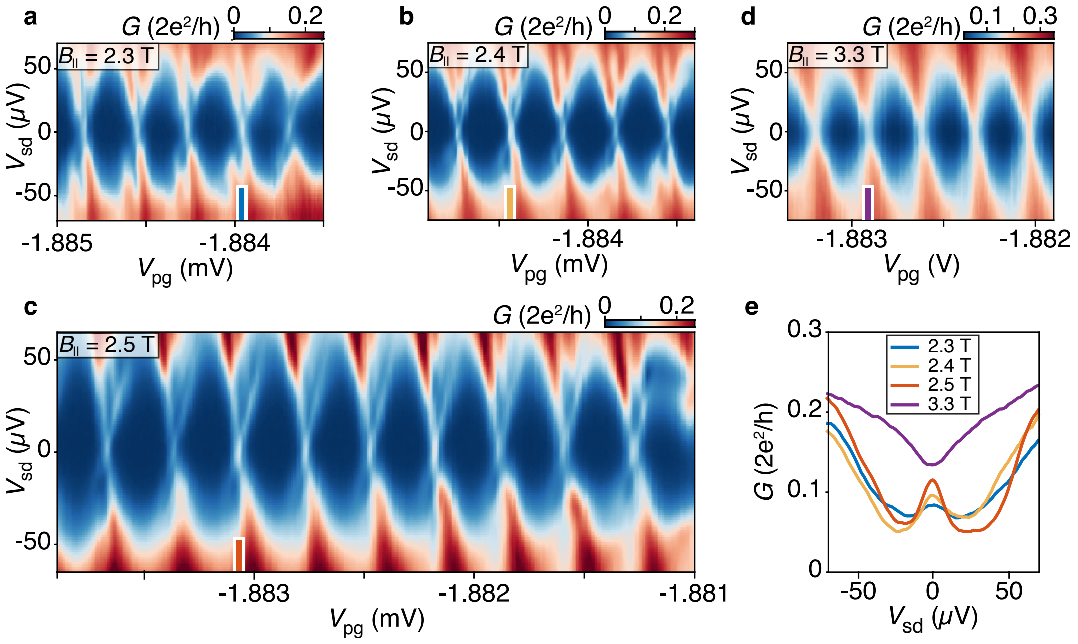

Supplementary Figure 2. **Stability of the zero-bias state c-f**, Differential conductance G as a function of V_{sd} and V_{pg} showing Coulomb diamonds for $B_\parallel = 2.3$ T (**a**), 2.4 T (**b**), 2.5 T (**c**), and 3.3 T (**d**). **e**, Line cuts of G vs V_{sd} at charge degeneracy showing a discrete zero bias peak in the $1e$ regime and a zero-bias dip in the normal state. The measurements were taken in the same gate configuration as Fig. 1 in the main text

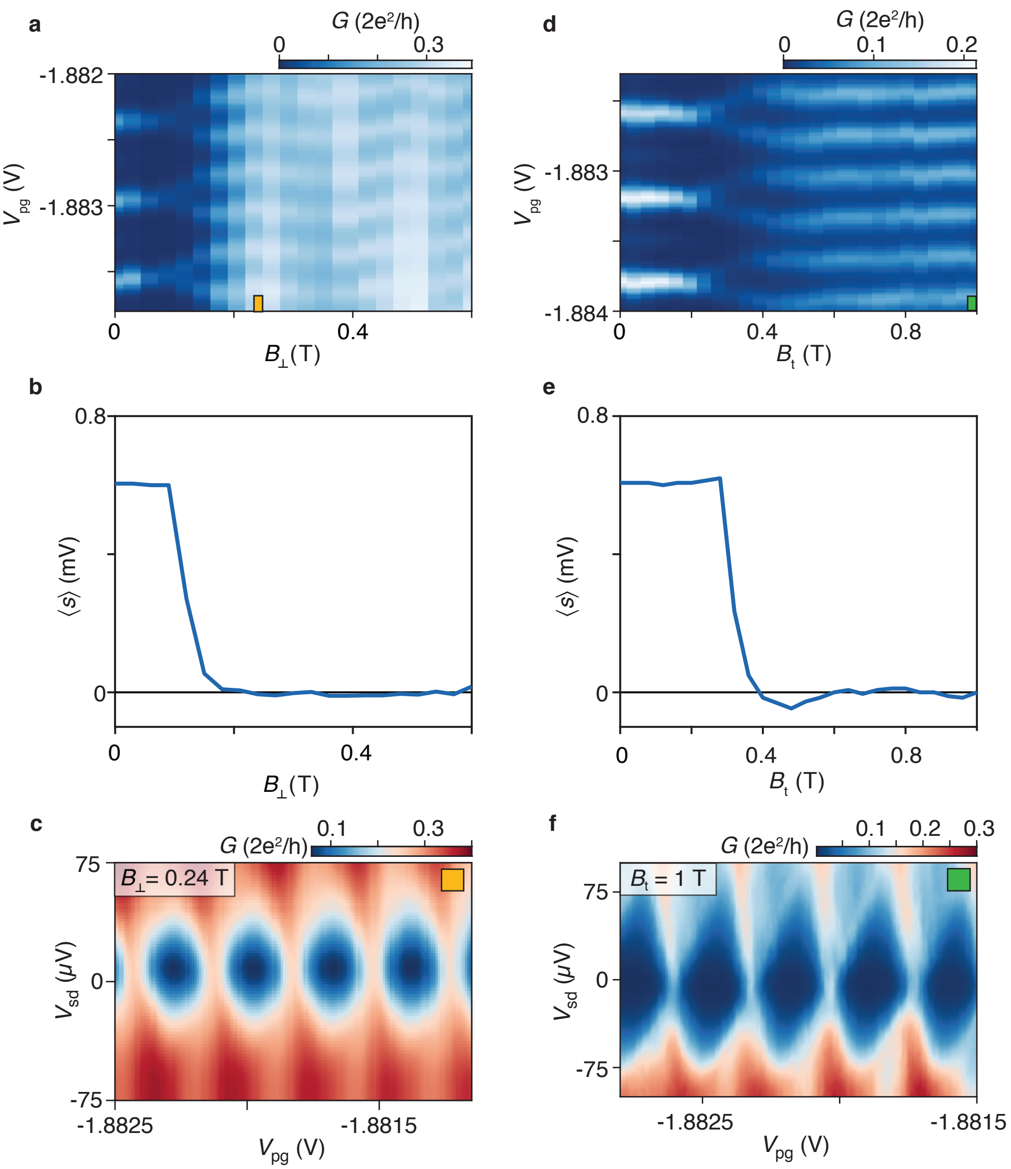

Supplementary Figure 3. **Transverse and perpendicular fields for device 1 in the regime of Fig. 1 of the main text**. **a**, Zero-bias differential conductance G as a function of gate voltage $V_{\rm pg}$ controlling electron occupancy and perpendicular field, $B_\perp$. **b**, CB peak spacing difference $\langle S \rangle$ as a function of $B_\perp$. **c**, Differential conductance G as a function of source-drain bias voltage $V_{\rm sd}$ and $V_{\rm pg}$ showing Coulomb diamonds for $B_\perp = 0.24$ T. **d**, Zero-bias differential conductance G as a function of $V_{\rm pg}$ and transverse field, $B_{\rm t}$. **e**, CB peak spacing difference $\langle S \rangle$ as a function of $B_{\rm t}$. **f**, Differential conductance G as a function of $V_{\rm sd}$ and $V_{\rm pg}$ showing Coulomb diamonds for $B_{\rm t} = 1$ T. The field directions are represented in Fig. 1a in the main text.

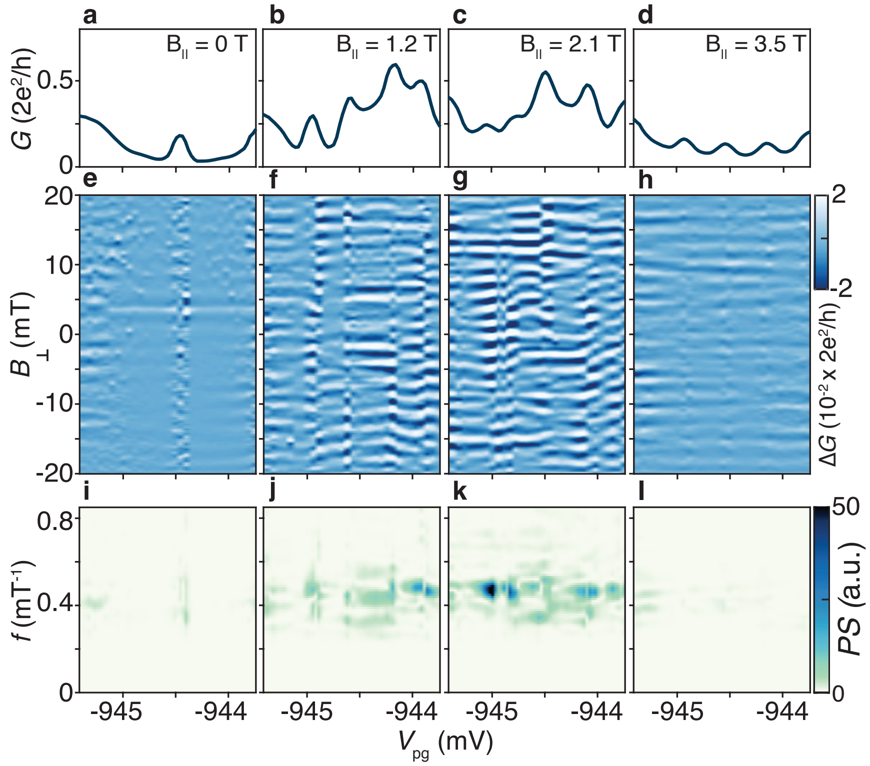

Supplementary Figure 4. **Conductance oscillations evolutions in parallel field for Device 2**. Magnetoconductance for parallel field values $B_{\parallel} = 0$, 1.2, 2.1, and 3.5 T (left to right). **a-d**, Zero-bias differential conductance $G(B_{\perp} = 0)$ versus gate voltage $V_{\rm pg}$ controlling electron occupation. **e-h**, Magnetoconductance ΔG as a function of $V_{\rm pg}$ and perpendicular field $B_{\perp}$ controlling the flux in the interferometer with corresponding power spectra in **i-l**. A single flux quantum piercing the loop area $A_{\rm loop} \sim 1.8$ μm^2 corresponds to a frequency $f_{\rm loop} = A_{\rm loop}/(h/e) \sim 0.44$ mT^{-1}. **e-l**, a slowly varying background has been subtracted.

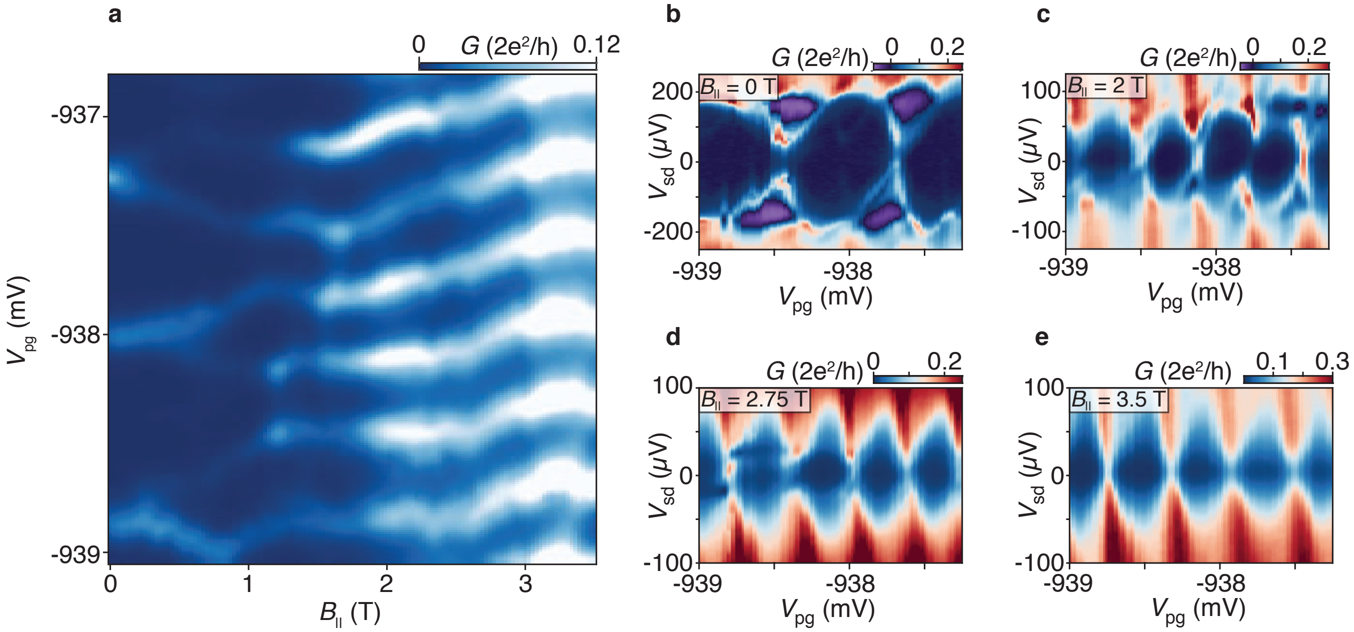

Supplementary Figure 5. **Coulomb blockade for device 2. a**, Zero-bias differential conductance G as a function of parallel magnetic field $B_{\parallel}$ and gate voltage $V_{\rm pg}$ controlling the electron occupancy with the reference arm closed. **b-e**, Differential conductance G as a function of voltage bias $V_{\rm sd}$ and $V_{\rm pg}$ for $B_{\parallel} = 0$ T (**b**), 2 T (**c**), 2.75 T (**d**), and 3.5 T (**e**).

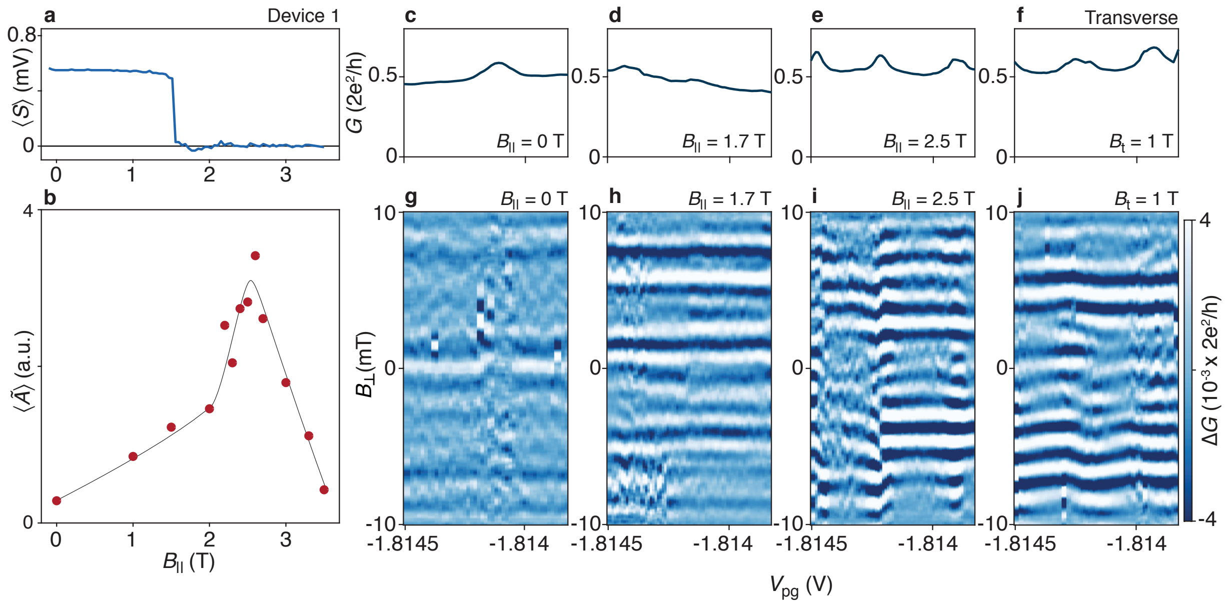

Supplementary Figure 6. **Second gate configuration of device 1**. **a**, Peak spacing difference $\langle S \rangle$ as a function of parallel magnetic field $B_{\parallel}$. **b**, Aharonov-Bohm oscillation amplitude $\langle \tilde{A} \rangle$ as a function of $B_{\parallel}$. The solid line is a guide to the eye. **c-j**, Magnetoconductance for parallel magnetic fields $B_{\parallel} = 0$ T, 1.7 T, and 2.5 T and transverse magnetic field $B_{\mathrm{t}} = 1$ T (left to right). **c-f**, Zero-bias differential conductance $G(B_{\perp} = 0)$ versus gate voltage V_{pg} used to control electron occupation. **g-j**, Magnetoconductance ΔG as a function of V_{pg} and perpendicular magnetic field $B_{\perp}$ controlling the flux in the interferometer.

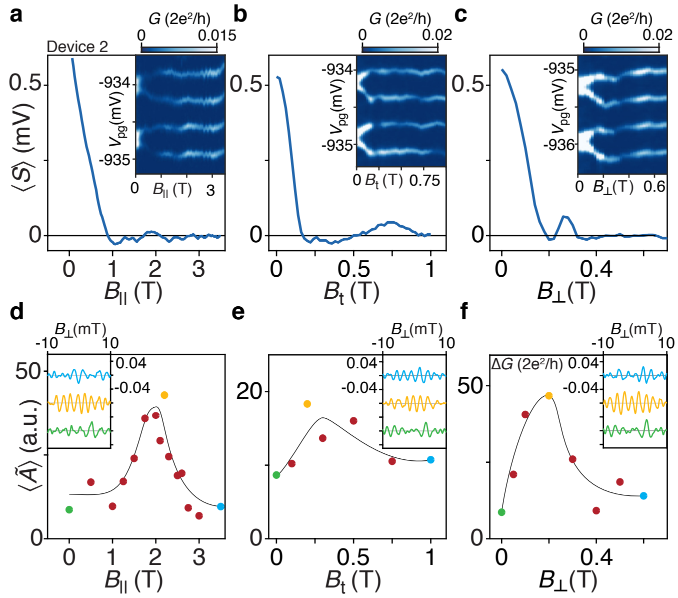

Supplementary Figure 7. **Orthogonal magnetic fields.** **a-c**, Peak spacing difference $\langle S \rangle$ as a function of magnetic fields $B_{\parallel}$, B_{t}, and $B_{\perp}$ for **a**, **b**, and **c**, respectively. Insets show the zero-bias differential conductance G as a function of magnetic field and gate voltage V_{pg} controlling electron occupancy with the reference arm closed. **d-f**, Oscillation amplitude $\langle \tilde{A} \rangle$ as a function of magnetic fields $B_{\parallel}$, B_{t}, and $B_{\perp}$ for **d**, **e**, and **f**, respectively. The solid lines are a guide to the eye. Insets show magnetoconductance ΔG traces as a function of small perpendicular magnetic field $B_{\perp}$. Curves with the largest AB oscillation amplitude are shown for specific magnetic fields indicated by the marker color in the main panel.

[1] Ludwig, T. & Mirlin, A. D. Interaction-induced dephasing of Aharonov-Bohm oscillations. *Phys. Rev. B* **69**, 193306 (2004).
